# Between-days intra-rater reliability with a hand held myotonometer to quantify muscle tone in the acute stroke population

**DOI:** 10.1038/s41598-017-14107-3

**Published:** 2017-10-26

**Authors:** Wai Leung Ambrose Lo, Jiang Li Zhao, Ling Chen, Di Lei, Dong Feng Huang, Kin Fai Tong

**Affiliations:** 1 Department of Rehabilitation Medicine, Guangdong Engineering and Technology Research Center for Rehabilitation Medicine and Translation, The First Affiliated Hospital, Sun Yat-sen University, Guangzhou, 510080 China; 2 Department of Electronic and Electrical Engineering, University College London, Torrington Place, London, WC1E 7JE England

## Abstract

A myotonometer can objectively quantify changes in muscle tone. The between-days intra-rater reliability in a ward setting for the acute stroke population remains unknown. This study aimed to investigate the device’s between-days intra-rater reliability when used in a ward setting for acute stroke participants. Muscle tone of biceps brachii, brachioradialis, rectus femoris, and tibialis anterior was recorded in the ward at bedside by one physiotherapist on two consecutive days. This study included participants who were within 1 month of their first stroke occurrence. Participants who were medically unstable or who suffered from brain stem injury were excluded. Reliability was assessed by the intraclass correlation coefficient (ICC), standard error of measurement (SEM), smallest real difference (SRD), and the Bland-Altman limits of agreement. The results indicated excellent between-days intra-rater reliability (ICC > 0.75). SEM and SRD show small differences between measurements. The Bland-Altman analysis indicated a tendency of overestimation of the rectus femoris. MyotonPRO demonstrated acceptable reliability when used in a ward setting in those patients with acute stroke. However, results should be interpreted with caution, due to the limitations of the study and the varying level of consistency observed between different muscles.

## Introduction

Stroke is among the leading causes of disability worldwide^1^. The most common muscular impairment following stroke is spasticity^2^, which affects muscle tone. Muscle tone is considered fundamental in maintaining balance, postural stability, and energy-efficient muscle contractions^3^. Part of the rehabilitation goal is to restore normal muscle tone to enable normal motor function and reduce pain. The Ashworth scale or the Modified Ashworth Scale are the most commonly used instruments to assess muscle tone in clinical and research settings^4^. These scales have been criticized for their subjective limitations, lack of validity and reliability^5,6^. Standardized techniques such as ultrasound imaging with dynamometry^7^ and magnetic resonance elastography^8^ are not always clinically feasible. Consequently, there is no valid, reliable, and practical instrument that can record muscle tone in a clinical setting^9^. Given that the prevalence of strokes are rising every year^10^, there is an urgency to identify a reliable method to quantify changes in muscles tone in stroke patients.

MyotonPRO (Myoton, Ltd., London, UK) is a device that can quantify changes in muscle tone. The technology has been around for over a decade. The latest model has an embedded triaxle accelerometer that allows the device to be held in any direction when taking measurements, enabling different postures and position^2^. This should theoretically improve its clinical use in a ward setting where there may be less room for therapists to maneuver into the ideal position, or for patients with reduced mobility such as patients those with acute stroke.

The reliability of the technology has been assessed in a healthy population^2,11–13^, in patients with Parkinson’s disease^14^, or with subacute and chronic stroke^15,16^ and in the elderly with paratonia^17^. The majority of published studies demonstrated high intra-rater reliability within the same session and between-days, particularly in the healthy population when measurements were done in a laboratory setting. However, the latest published study^17^ indicated that the intra-rater reliability on MyotonPRO decreased substantially in the elderly with paratonia. Between-days intra-rater reliability was more affected than within-day intra-rater reliability. This finding indicated that results from one population may not necessarily be generalized to other populations. Only two studies were found that had investigated the reliability of a previous model (Myoton-3) in patients with subacute and chronic stroke^15,16^. All published reliability studies were conducted in laboratory settings. The reliability of MyotonPRO, when used in a clinical setting, must first be established if it was to be recommended as a clinical outcome measure to monitor the effects of interventions.

To date, we found no study that investigated the between-days intra-rater reliability of MyotonPRO when used on a ward to record muscle tone in patients with acute stroke. The aims of this study were to assess the between-days relative and absolute intra-rater reliability of MyotonPRO used in a ward setting.

## Methods

### Recruitment

Participants were recruited from the inpatient rehabilitation ward of a university affiliated hospital. The study was part of a randomized controlled trial that investigated the effect of multisensory interactive training in patients with acute stroke. All consecutively admitted stroke patients were screened by members of the clinical team for eligibility as part of a routing clinical assessment. Potential participants were provided with written information about the study. A member of the research team approached them to inquire if they were interested and willing to take part in the study. A screening log of all non-recruited patients and reasons for exclusion were maintained.

### Sample population

Inclusion criteria were as follow: 1) within 1 month of the first occurrence of stroke; 2) stage 2 or above on the Brunnstrom classification at the upper extremity, the hand, or the lower extremity; 3) MRI or CT confirmed stroke; 4) ages between 40 to 80; 5) able to walk at least 10 meters with or without assistance; 6) no severe cognitive impairment (defined as Mini-Mental State Examination score as less than 10^18^). Patients who were medically unstable or were suffering from brain stem injury were excluded.

### Study setting

Measurements were taken in the ward at bedside by one physiotherapist on two consecutive days between 3 pm to 5:30 pm. A one day period was chosen to minimize impact on reliability due to changes of muscle tone related to intervention or natural recovery. Previous study that tested the intra-rater agreement of the modified Ashworth scale on acute stroke patients used a one day interval^19^. The authors concluded that if a patient was measured, or had an intervention and was then re-measured, it was more likely that the observed difference between the two measurements were due to unreliability of the measurement as opposed to the effect of intervention. The assessor in this study was a 34-year-old female stroke specialist physiotherapist at the local institute. She was educated with a master’s degree and had 10 years of clinical experience at the time of data collection. The assessor underwent 4 hours of training with the manufacturer’s representative, which included theory and practical aspects of operating the device. This was followed by 16 hours of practice with another research physiotherapist who had prior experience with the device. The sessions involved practicing test site identification, data recording, and analysis on healthy individuals (n = 5) and on patients (not including participants recruited in this study) in the ward (n = 5).

### Ethics

The Medical Ethical Committee of the First Affiliated Hospital of Sun Yat-sen University (Ethics No. [2014]88) approved the study protocol. The study was conducted in accordance with the relevant guidelines and regulations of the local institute. All patients who met the criteria were invited to take part in the study. They were given time to consider whether they wished to take part in the trial and ask any questions prior to data collection. Written informed consent was obtained from all patients who agreed to take part. All participants could withdraw from the trial at any time without giving a reason.

### Equipment

MyotonPRO was used to measure muscle tone of the upper and lower extremities on the affected side. The test end of the MyotonPRO was placed perpendicularly to the middle of the tested belly muscle to avoid muscle tone being affected by gravity. The probe was pushed against the skin to the required depth. Triple scan mode consisted of a set of three consecutive impulses, one second apart in this study. After each set of measurements, the values for mean, standard deviation (SD), and coefficient of variation (CV) for the three measurements were displayed on the screen. As standard operating procedure recommended by the manufacturer, any measurement set with a CV over 3% was erased and re-measured.

### Parameters

The technology measures muscle tone by applying multiple short impulses over the muscle bulk via the testing probe^20^. The transducer records the acceleration of muscle oscillation response and calculates the oscillation frequency (Hz). The oscillation frequency characterizes muscle tone and is calculated as Hz = 1/T, where T is the oscillation period in seconds.

### Procedure

The studied muscle groups were: 1) biceps brachii, 2) brachioradialis, 3) rectus femoris, and 4) tibialis anterior. Measurement began on the left, and progressed to the right. Two sessions took place at a similar time on two consecutive days. These muscles were chosen as they were validated in previous studies^9,14,21,22^ and has an important role in activities of daily living and functional mobility^23–25^.

Participants were in a supine lying position, elbow flexed to 10–15°, forearm supinated with a towel placed under the wrist when taking a measurement of the biceps brachii. A measuring tape was used to locate the testing site, which was the half way point between the anterior aspect of the lateral tip of the acromion and the medial boarder of the cubital fossa. A pen was used to mark the testing site which was left on the skin to guide the second measurement. The rationale behind using a pen to mark the test site was to minimize confounding factors related to repeatability of test site identification. This study specifically assessed the reliability of the device when used in a ward setting. When measuring the brachioradialis, the test site was marked by identifying the upper two-thirds distance from the lateral supracondylar ridge to the styloid process (when the elbow was extended and forearm pronated). When measuring the rectus femoris, patients were positioned in supine lying with their hips in a neutral position and the knees fully extended. The testing site was measured at two thirds of the distance between the anterior superior iliac spine and superior pole of the patellar. Measurements of the tibialis anterior were taken at the upper two thirds of the distance between the lateral condyle of the tibia to the medial cuneiform.

Blinding of the assessor was attempted by removing the data from the device after the first measurement, so that the assessor could not revisit the first set of readings. The operating procedure should minimize the effect of memory bias, as the operator is only required to place the device at the test location. The device then records the parameters with minimal input from the assessor.

### Data analysis

Statistical analyses were performed using SPSS 20 software (IBM, Armonk, NY, US). Data normality was assessed by the Kormongorov-Shminov test and frequency histograms. The characteristics of the sample population included age, gender, body weight, height, affected side, and onset days since the stroke were assessed by descriptive statistics.

Intraclass correlation coefficient (ICC) was used to determine the relative reliability. The ICC model of ICC (3, k) was used to assess the relative intra-rater reliability, with an ICC value of over 0.75 suggested to be excellent^26–28^. There are various classification scales that exist for interpreting level of reliability from ICC. This study interprets the ICC level based on the recommendation by Fleiss^29^: Excellent ICC > 0.75; Good to Fair = 0.74–0.40; Poor < 0.40.

Hopkins^30^ refers absolute reliability, such as the standard error of measurements, as the “typical error” that would be expected. Absolute reliability indices give actual measurements values or ranges in the same units of measurement that can be interpreted clinically^31^. Standard error of measurements (SEM), smallest real difference (SRD)^32^ and 95% limits of agreement (LOA)^33^ were the absolute reliability indices used in this study. Systematic bias was identified by Bland and Altman analysis^34^. The differences between the first and second measurements were assessed by a paired t-test (*p* < 0.05).

### Data Availability

The data sets generated and/or analyzed during the current study are available from the corresponding author based on a reasonable request.

## Results

Twenty-eight participants with acute stroke were recruited for this study from a single center. The mean age of the sample population was 58. The characteristics of the sample population are provided in Table 1. Descriptive statistics of the myotonometric measurements in two sessions are provided in Table 2. Medical records show that none of the participants were on medication in order to control spasticity.

**Table 1:** Demographics data of all participants.

*Characteristic*	*Value*
Age (mean, SD)	58.7 (12.8)
Dominant side, left/right	0/28
Affected side, left/right	13/15
Gender, male/female	24/4
Body Mass Index (mean, SD)	22 (4)
Days after stroke onset, (mean, SD)	22 (7.36)
***Brunnstrom classification*** (***median***, ***mode***)	
Upper extremity	3 (2)
Hand	2 (1)
Lower extremity	3 (3)
***MAS*** (***median***, ***mode***)	
Biceps brachii	1 (2)
Brachioradialis	1(2)
Rectus femoris	1 (2)
Tibialis anterior	0 (1)

*Keys: SD – standard deviation; MAS – modified Ashworth scale.Relative reliability*.

**Table 2:** The between-rater mean difference and ICC index.

Relative reliability					
Muscle	Variable	First measurement	Second measurement	d	ICC (95% CI)
Biceps brachii	Tone (Hz)	13.07 (1.14)	13.28 (1.90)	−0.21	0.75 (0.49–0.89)
Brachioradialis	Tone (Hz)	16.92 (1.82)	16.11 (1.66)	0.81	0.82 (0.47–0.93)
Rectus femoris	Tone (Hz)	15.11 (2.50)	14.69 (2.16)	0.42	0.81 (0.58–0.91)
Tibialis anterior	Tone (Hz)	18.38 (2.50)	18.84 (2.35)	−0.46	0.81 (0.37–0.86)

*Key: d – mean difference between first and second measurements*.

### Relative reliability

The ICCs of the four studied muscles are 0.75 or above, which suggest excellent reliability. The confidence intervals (CIs) of the four studied muscles are wide, with the lower bound below the acceptable level. A summary of the ICCs and CIs is presented in Table 2.

#### Absolute reliability

A summary of the results on absolute reliability is provided in Table 3. SEM and SRD of lower extremities were higher than upper extremities. The highest SEM and SRD were observed at tibialis anterior.

**Table 3:** Absolute reliability indices.

Muscles	Variable	SEM	SRD
Biceps brachii	Tone (Hz)	0.76	2.41
Brachioradialis	Tone (Hz)	0.50	1.96
Rectus femoris	Tone (Hz)	0.83	2.52
Tibialis anterior	Tone (Hz)	1.24	3.08

Key: SEM – standard error measurement, SRD-smallest real difference.

#### Bland and Altman analysis

**Figure 1:**
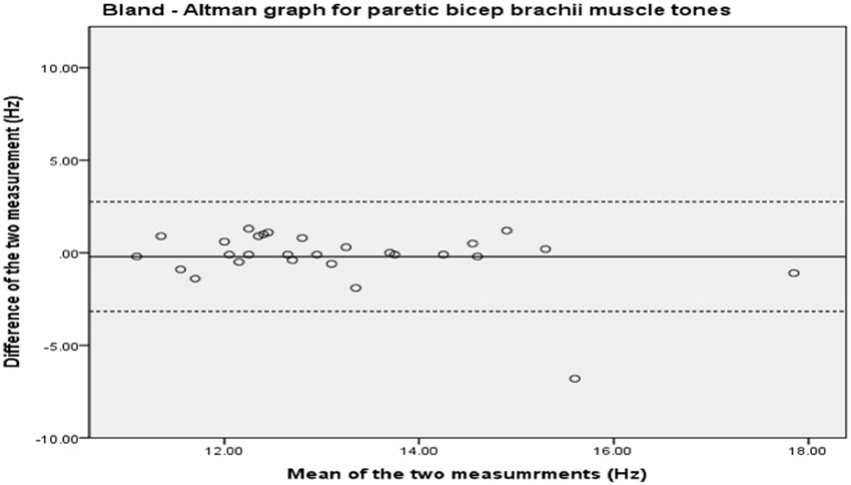
Bland-Altman analysis plot for biceps brachii.

The Bland-Altman plot for biceps brachii (Fig. 1) shows no systematic bias, as the data points distribute equally below and above the mean. The second measure appears to underestimate at high muscle tone, with an outlier at the second highest mean muscle tone. The 95% limits of agreement are +2.76 to −3.17.

**Figure 2:**
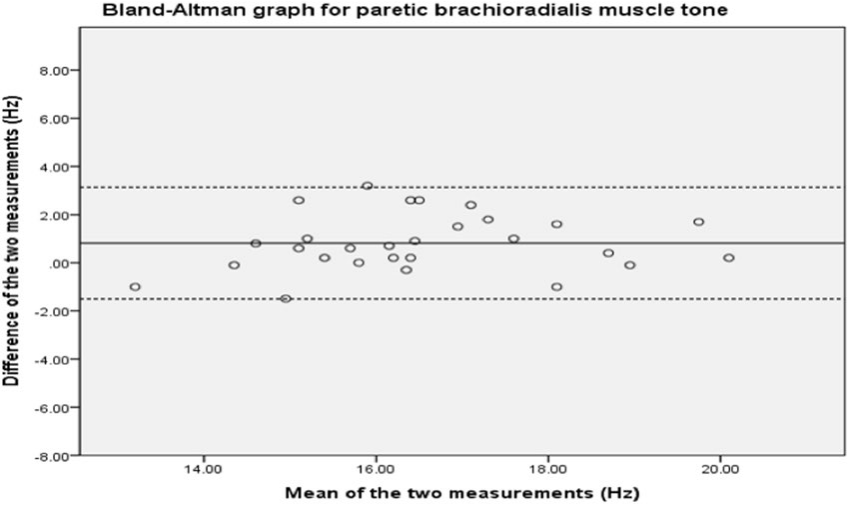
Bland-Altman analysis plot for brachioradialis.

The Bland-Altman plot for brachioradialis (Fig. 2) shows no systematic bias between the first and second measurements. The 95% limits of agreement are −1.51 to +3.4.

**Figure 3:**
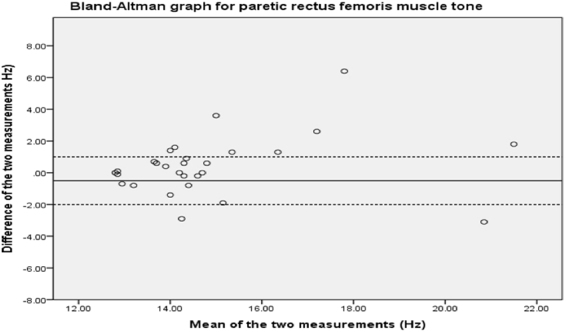
Bland-Altman analysis plot for rectus femoris.

For rectus femoris, the Bland & Altman plots (Fig. 3) show bias towards overestimating muscle tone on the first measurement, as data points are above the mean difference and upper limits of agreement. The 95% limits of agreement are −3.25 to +4.09.

**Figure 4:**
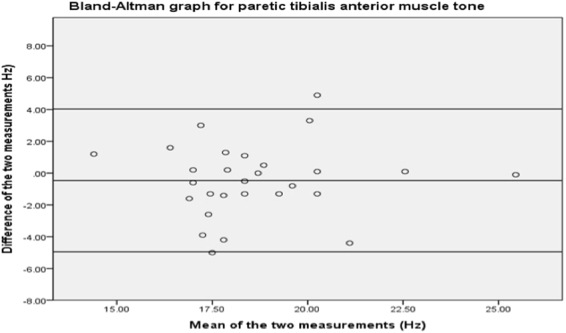
Bland-Altman analysis plot for tibialis anterior.

Figure 4 shows no systematic bias between the first and second measurements on the tibialis anterior. The 95% limits of agree agreement are −4.95 to +4.03.

## Discussion

This study was among the first that assessed the between-days intra-rater reliability on MyotonPRO when used in a ward setting of the acute stroke population. Our findings from this study provide data on the typical error and magnitude of agreement, to which clinicians can refer when using the device in a ward setting on people with acute stroke.

### Relative reliability

ICC is a single index which reflects both degrees of consistency and agreement among ratings^35^. The closer the value is towards 1, the higher the agreement and consistency between the two measurements. Some authors suggested any measure should have an ICC of at least 0.6 to be useful^36^ and some authors suggest that ICC of at least 0.75 could be considered to be excellent^27,29^. In the present study, the ICCs for the four studied muscles were 0.75 or above, which indicated excellent between-days intra-rater reliability. This observed reliability was higher than reported in the elderly with paratonia (ICC = 0.247)^17^ for biceps brachii. The difference between the two studies may be related to the difference in pathology. Van Deun *et al*. excluded neurological conditions^17^ whereas this study included participants who suffered from stroke. It was possible that the device was not as reliable when measuring muscle tone in a certain pathological group or in people with high muscle tone. The high between-days intra-rater reliability of the rectus femoris in this study was consistent with those recorded in healthy individuals^11^. These findings suggest that the device was reliable in recording muscle tone of the rectus femoris in the acute stroke population when used in a ward setting. Despite these positive results, the interpretation of ICC was not always straightforward, as there was neither a standard acceptable level of reliability using ICC, nor sufficient published data that could suggest whether these reliability levels were clinically acceptable with MyotonPRO or in an acute stroke population. This was further complicated by the fact that the majority of the literature on ICC cut-off values was related to inter-rater reliability. Inter-rater reliability could be expected to be lower than intra-rater reliability^37^. A higher ICC level should therefore be expected, potentially reaching 0.8–0.9.  The lower bounds of CIs for all tested muscles were below the level of acceptable reliability. In addition, the relatively wide CIs imply low statistical power due to small sample size^34^. Thus, no firm conclusion could be drawn from the ICC analysis. One of the criticisms of ICC was that it was likely to be affected by between-subject variability in the sample population^31,38^; thus, a low ICC could be found even when trial-to–trial variability was low if the between-subjects variability was low^39^. This variable may be a contributing factor to the low ICC value observed for biceps brachii, as it had the lowest standard deviation. Due to the ICC limitation, it was accompanied by absolute reliability indices to comprehensively assess reliability of the device.

### Absolute reliability–SEM and SRD

Absolute reliability refers to the degree on which repeated measurements vary for individuals^35^. SEM and SRD are expressed in actual units of measurement that enable comparison between studies. The SEM and SRD in this study for biceps brachii were smaller than those reported in the elderly with paratonia (SEM = 1.96 Hz, SRD = 5.45 Hz) and healthy elderly (SEM = 1.13 Hz, SRD = 3.14 Hz)^17^. This was similar to the finding of relative reliability, which in turn suggested the device was reliable at measuring muscle tone on different days in the acute stroke population.

The SEM and SRD of the rectus femoris muscle tone were slightly higher than those reported in the healthy population (a difference of 0.13 Hz and 0.62 Hz, respectively)^11^. The small error between measurements suggested that the between-days measurements taken in a ward on patients with acute stroke were consistent and reliable, but not as consistent when used in a laboratory on healthy individuals. Thus, a larger change would be required to be deemed “real” change. SEM and SRD were higher at the lower extremity than the upper extremity, indicating varying levels of reliability in different muscles. This was consistent with the study by Chuang *et al*.,^15^ who reported differences in SEM and SRD of 0.69 Hz and 1.83 Hz, respectively, between deltoid and biceps brachii in patients with chronic stroke as measured by the Myoton–3. Our findings indicate that reliability may vary between different muscle groups.

The interpretation of the SEM and SRD from this study should be cautious due to confounding factors of natural variation and spontaneous recovery which may contribute to the trial-to-trial variability. However, these confounding factors are difficult to control in people with acute stroke, as they are part of the pathology. Thus, any between-days reliability study regarding this population group is likely to be affected by these factors. Previous study by Gregson^19^ concluded that if a patient with acute stroke was measured, had an intervention and was then re-measured after one day, it was likely that the observed difference between the measurements were due to unreliability of the measurement. It could be argued that the amount of difference in SEM and SRD between these studies did not exceed the SRD values of published studies in healthy populations^11^, subacute stroke populations^15^, and elderly with paratonia^17^. The differences are therefore unlikely to be “real” differences.

### Bland-Altman analysis

To date, there are limited studies available for the comparison of the 95% limits of agreement. The purpose is to indicate the magnitude of disagreement between measures, providing a range of error that may relate to clinical acceptability^35^. The Bland-Altman plot is a visual presentation that allowed identification of systematic bias. The present results indicate a tendency of overestimation of the first measurement when assessing rectus femoris. A possible reason for overestimation may be related to the natural recovery or variation of muscle tone. We found no evidence in our literature search that indicated how muscle tone varies on a day-to-day basis in the first month of stroke occurrence. However, given that altered muscle tone in the stroke population is a result of lesion at the cortex and hyperactivity of the spinal reflex^40^, it is unlikely that muscle tone could be affected in a short period. This study did not control physical activities of the participants on the days of data collection, which may contribute to variation in muscle tone estimation. However, it is well documented within literature^41^ that acute stroke patients are generally inactive during their stay in ward. Thus, it is unlikely that participants would have large difference in activity levels on two consecutive days.

No systematic bias was observed for all other studied muscles, which suggested consistency between the two measurements. The range of the 95% limits of agreement for the quadriceps was wider in this study than previously reported^11^. This suggests that using the device in a ward setting between-days was not as consistent as using it in the laboratory. These studies observed a wide range of 95% limits of agreement among the studied muscles. The variation in consistency between studied muscle groups was similar to the findings of ICC, SEM, and SRD of the present study. The difficulty in interpreting the results of the Bland and Altman analysis is that there is no universally accepted range of limits of agreements. To date, there is limited study that indicates if the observed range of error is clinically acceptable as no other study reported intervention-induced quantitative changes of muscle tone measured by myotonometer. Findings from the present study form the reference for measuring changes of muscle tone on different days.

### Limitations

Physical activities between the two measurements were not controlled. This may affect the recording of muscle tone on separate days. The relaxation state and anxiety state at the time of data collection were also not objectively recorded by other means. Thus, it could not be certain that the studied muscles were in a resting state or at a comparable state during the two recording sessions. This may contribute to the underestimation of reliability. This study did not specifically set out to test reliability of the device on a range of participants with different spasticity levels or at different stages of the Brunnstrom scale. Although by doing so would increase the generalizability of the current findings, the primary aim of this study was to establish intra-rater reliability of the device when used in a ward setting for those with acute stroke. The sample size of the study may be considered small, which affects the power of the statistical analysis and prevents us from drawing a firm conclusion.

### Further research

Further research is recommended to test the reliability of the device when used in patients with a range of spasticity in a ward setting. It is necessary to assess inter-rater reliability with a larger sample population, along with therapists to determine whether measurements can be generalized across physiotherapists with sufficient statistical power. New research should also assess the validity of the myotonometer measurement against accepted instruments, such as the Ashworth Scale, in a ward setting. This would provide information on the construct validity of both instruments and their interchangeability.

## Conclusions

This present study demonstrates that MyotonPRO has acceptable between-days intra-rater reliability in measuring muscle tone in a ward setting. Agreement between measurements is acceptable, while measurement error is comparable with the existing literature. One must be cautious when interpreting the results due to the limitations of the study.

### Declarations

#### Ethical Approval and Consent to participate

The study was approved by the Medical Ethical Committee of the First Affiliated Hospital of Sun Yat-sen University (Ethics No. [2014]88). Written consent was obtained from all patients who agreed to take part in the study.

### Consent to Publish

We obtained written informed consent from participants for publication of their individual details and images in this manuscript. The consent form is held by the authors’ institution and can be reviewed by the Editor-in-Chief.
